# Comorbidity in Older Patients Hospitalized with Cancer in Northeast China based on Hospital Discharge Data

**DOI:** 10.3390/ijerph17218028

**Published:** 2020-10-31

**Authors:** Xiao-Min Mu, Wei Wang, Fang-Yi Wu, Yu-Ying Jiang, Ling-ling Ma, Jia Feng

**Affiliations:** 1 Department of Medical Informatics, School of Public Health, Jilin University, Changchun 130021, China; muxm18@mails.jlu.edu.cn (X.-M.M.); w_w@jlu.edu.cn (W.W.); yyjiang18@mails.jlu.edu.cn (Y.-Y.J.); mall19@mails.jlu.edu.cn (L.-L.M.); 2 Information Research Center of Military Sciences, Academy of Military Sciences, Beijing 100039, China; wufy16@mails.jlu.edu.cn

**Keywords:** cancer, comorbidity, association rule, prevalence ratio

## Abstract

Patients with cancer often carry the dual burden of the cancer itself and other co-existing medical conditions. The problems associated with comorbidities among elderly cancer patients are more prominent compared with younger patients. This study aimed to identify common cancer-related comorbidities in elderly patients through routinely collected hospital discharge data and to use association rules to analyze the prevalence and patterns of these comorbidities in elderly cancer patients at different cancer sites. We collected the discharge data of 80,574 patients who were diagnosed with cancers of the esophagus, stomach, colorectum, liver, lung, female breast, cervix, and thyroid between 2016 and 2018. The same number of non-cancer patients were randomly selected as the control group and matched with the case group by age and gender. The results showed that cardiovascular diseases, metabolic diseases, digestive diseases, and anemia were the most common comorbidities in elderly patients with cancer. The comorbidity patterns differed based on the cancer site. Elderly patients with liver cancer had the highest risk of comorbidities, followed by lung cancer, gastrointestinal cancer, thyroid cancer, and reproductive cancer. For example, elderly patients with liver cancer had the higher risk of the comorbid infectious and digestive diseases, whereas patients with lung cancer had the higher risk of the comorbid respiratory system diseases. The findings can assist clinicians in diagnosing comorbidities and contribute to the allocation of medical resources.

## 1. Introduction

The incidence of cancer (and cancer mortality rates) is rising rapidly worldwide due to the aging population [1]. Cancer is the leading cause of death and produces a heavy disease burden in China [2]. It has been reported that more than one-half of patients with cancer >65 years of age often carry the dual burden of cancer itself and other co-existing chronic conditions [3,4]. Individuals have multiple medical conditions referred to as comorbidity [5]. Comorbidities potentially affect the stages of the cancer spectrum from diagnosis, through treatment, to outcome [6]. Patients with comorbidity are substantially more likely to experience complicate treatment, increased cost of care, decreased quality of life and lower survival probabilities than those without comorbidity [3,7]. Therefore, understanding cancer comorbidities can help to comprehend the pathogenesis of comorbidities, promote the prevention and control of comorbidities, and assist the health administration department to rationally evaluate the status of comorbidities to better optimize the distribution and utilization of medical resources.

Many population-based surveys and clinical studies have attempted to explore the prevalence of comorbidities and the impact of comorbidities on health care or survival outcomes, such as identifying the comorbidity patterns of mental diseases [8,9], and assessing the impact of comorbidity on health care or outcomes of chronic diseases in the elderly [10,11]. Researchers have attempted to determine the risk of comorbidities in cancer patients [3,12], focusing on specific disorders related to cancers, such as cardiovascular and cerebrovascular diseases [13,14], hypertension [15,16], other associated complications, and/or specific populations with cancer, such as the elderly [17,18]. The overall pattern of cancer comorbidities has not been established to date because survey data are relatively small in size, usually focus on specific disorders, and sometimes include inadequate information on diagnosis and treatment. Therefore, there is a need for comprehensive information from large long-term datasets to improve our understanding of the prevalence of cancer-related comorbidities and analyze the comorbidity patterns.

With the development and advances in information technology, the emergence of electronic medical record (EMR) systems has made it possible to use clinical information for disease relationship mining. Hospital discharge data, as a type of administrative data derived from EMR, are becoming one of the available data sources for assessing disease comorbidities [19,20], with the discharge diagnosis codes assigned by trained physicians following standard guidelines. Therefore, the use of EMR data for comorbidity analysis has gradually attracted the attention of researchers, such as in identifying important comorbidities among cancer [21], analyzing the impact of comorbidities on cancer care and outcomes [22], and assessing comorbidities of substance abuse [23,24]. Most previous studies used statistical analysis methods, such as relative risk and φ-correlation, to mine comorbidity patterns. However, both of these measures mainly consider pairwise relationships, which cannot demonstrate all of the comorbid associations [25]. To completely detect the co-occurrence relationships, association rule mining [26] (ARM) was used in the current study to identify the comorbidity patterns of cancer patients. ARM is an important data mining technology that is used to mine the association between valuable data items from a large amount of data [25,27]. ARM makes it possible to analyze the association between not only two diseases, but also among three or more comorbidities that can be calculated from existing statistics.

In this study, we used hospital discharge data derived from 16 tertiary hospitals in northeast China between 2016 and 2018 to identify important cancer-related comorbidities and estimate the prevalence and patterns of these comorbidities among elderly cancer patients with diverse cancer sites.

## 2. Materials and Methods

### 2.1. Study Population and Data Source

The 10th revision of the International Classification of Diseases (ICD-10) [28] is used in the public hospital diagnosis system of Jilin Province. All categories of comorbidities in the current study followed the original categories of the ICD-10 system. The selection of cancers for this study included cancers of the esophagus (ICD 10 code C15), stomach (ICD 10 code C16), colorectum (ICD 10 codes C18-C20), liver (ICD 10 code C22), lung (ICD 10 code C34), female breast (ICD 10 code C50), cervix (ICD 10 code C53), and thyroid (ICD 10 code C73), which are the predominant malignancies based on incidence and mortality [29]. Jilin Province is located in northeast China and had 27.04 million permanent residents in 2018. Because of environmental factors and dietary habits, such as serious air pollution, a love of pickled cabbage, smoking and drinking, northeast China has a high incidence of cancer, and the incidence of respiratory, digestive, and reproductive tract cancers ranks among the highest in China. The data used in this study were obtained from the hospital discharge medical records of patients > 60 years of age who were diagnosed with the above cancers in Jilin Province, China between 2016 and 2018. In addition, we randomly selected 80,574 elderly patients without cancer as the control group, which was matched to the case group by age and gender.

The hospital discharge medical records included the following data: demographic, such as age and gender; disease diagnosis; and medication. The diagnostic data consisted of one primary diagnosis and up to 15 secondary diagnoses, which were coded by trained coders using ICD 10. We used demographic and diagnostic data to analyze the co-morbid relationships to cancers. To ensure data quality, we only included the medical records of patients from tertiary hospitals. Ethical approval to conduct this study was obtained from the Ethics Committee of the School of Public Health at Jilin University (Jilin, China) (grant number: ethical review 2020-02-01).

### 2.2. Statistical Analysis

The characteristics of patients were summarized using frequency distributions and proportions. We used quartiles to describe the number of comorbidities in patients, presented as median (interquartile range (IQR)). The prevalence ratios (PRs) and 95% confidence intervals (CIs) for categories of co-morbid diseases were calculated, which were based on the categories of ICD 10. Distributions of categories of co-morbid diseases were compared for cancer and non-cancer patients using chi-square tests. Differences were considered significant if the *p*-value was ≤ 0.01.

We adopted the Apriori algorithm, which is the best-known ARM algorithm to extract and analyze the patterns of liver cancer comorbidities. The Apriori algorithm is a frequent itemset algorithm for mining association rules. The association rules are evaluated by support (the number of occurrences of disease A and disease B among all patients) and confidence (the number of occurrences of disease A co-occurring with disease B). The formulas for support and confidence are presented below.

$$\mathrm{Support}\left(X\to Y\right)=\frac{Number\;of\;patients\;with\;X\;and\;Y}{Total\;number\;of\;patients}\;\mathrm{Confidence}\left(X\to Y\right)=\frac{Number\;of\;patients\;with\;X\;and\;Y}{Number\;of\;patients\;with\;disease\;X}$$

The basic premise of the algorithm is to first find all frequency sets, the frequency of which is at least as frequent as the pre-defined minimum support, then generate strong association rules from the frequency sets that satisfy the minimum support and minimum confidence. The advantage of the Apriori algorithm is that the structure is simple, easy to understand, and there is no complicated derivation, which greatly improves the efficiency of the algorithm. The result of the algorithm is a list of patterns between two sets of diseases in the form of “X→Y,” which indicates that if disease X exists, disease Y co-exists. Although each pattern is directed with an arrow, it does not mean causation between diseases, but only represents co-occurrences. To avoid confusion, we ignored the directions of the patterns, and considered all diseases in set X and Y to be associated. Herein, support > 0.01 and confidence > 0.5 were used according to performance of validation diseases.

## 3. Results

### 3.1. Patient Statistics

**Figure 1:**
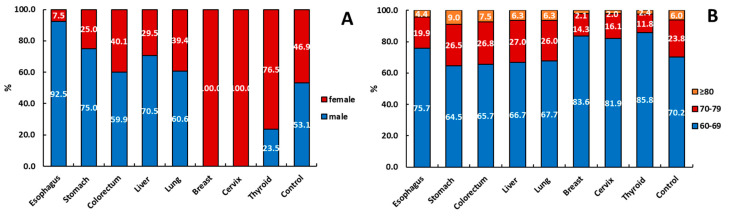
The age and gender distribution in the cancer and non-cancer study groups. (**A**) Gender distribution; (**B**) age distribution.

Figure 1 and Table 1 presents the age, gender, and number of comorbidities in the cancer and non-cancer study groups, each of which was comprised of 80,574 patients. There were 80,574 patients shown to have one of the specific cancers, as follows: esophagus, 3088; gastric, 8220; colorectal, 16,961; liver, 8710; lung, 28,282; breast, 11,231; cervix, 2623; and thyroid, 1459. Except for malignant tumors of the reproductive system, thyroid cancer patients were more likely to be female, while cancer patients with cancers other than thyroid cancer were more likely to be male; the distributions were consistent with the expected results. The patients in the cancer and non-cancer study groups were stratified using the following age brackets (in years): 60–69; 70–79; and ≥ 80. The largest age group was 60–69 years, and the proportion of each type of cancer was > 60%. In addition, the same analysis was performed on the control group.

As a group, the cancer patients most often had three comorbidities (median, 3; IQR, 1–6) and the prevalence of comorbidities tended to be higher in the cancer groups compared to the controls (median, 2; IQR, 1–4). Patients with liver (median, 4; IQR, 2–6) and lung cancers (median, 4; IQR, 2–7) were more likely to have higher levels of comorbidities than patients with other cancers. Females with digestive system cancers had more comorbidities than males (median, 3; IQR, 1–5 vs. median, 2; IQR, 1–5). Overall, the number of comorbidities increases with age in patients with cancer. Specifically, we observed that with age, the number of comorbidities in patients with thyroid cancer increases (median, 2; IQR, 1–4 to median, 5; IQR, 2–8).

### 3.2. Comorbidity Prevalence and Prevalence Ratios

Table 2 lists the prevalence of comorbidities and PRs in elderly patients with and without cancer. Table 3 presents the PRs of co-morbid diseases for each type of cancer compared with the control group. We omitted cancer metastases because we considered metastases to represent an advanced stage of cancer rather than an independent comorbidity. The highest prevalences of comorbidities that existed among elderly cancer patients were circulatory system (35.11%), digestive system (29.60%), and metabolic diseases (24.20%). However, the PRs of the circulatory system and metabolic diseases in cancer patients were lower than that in non-cancer patients, and the same results were obtained when comparing all types of cancers. Infectious diseases, blood system diseases, respiratory diseases, digestive diseases, and symptoms, signs and ill-defined conditions exhibited higher PRs when comparing elderly patients with and without cancers. Higher PRs of the comorbid infectious diseases, blood system diseases and digestive diseases were found in patients with cancers, especially in esophageal cancer, stomach cancer, colorectal cancer, liver cancer and lung cancer. Comorbid respiratory diseases showed higher PRs in esophageal cancer and lung cancer.

### 3.3. Association Rule Results

**Figure 2:**
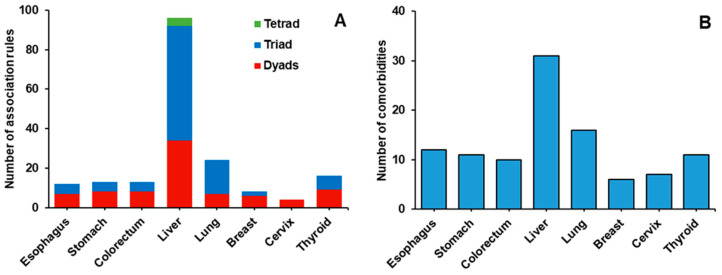
Basic properties of association rules. (**A**) Number of association rules; (**B**) number of comorbidities.

Figure 2 shows the number of rules and comorbidities for each cancer based on association rules analysis, which reflected the overall complexity of comorbidities in each type of cancer. The comorbidity pattern of liver cancer was the most complicated, with the largest number of comorbidities and association rules, followed by lung cancer. The number of comorbidities and association rules were similar for esophagus, stomach, and colorectum cancers. Cervix and breast cancers had the least number of comorbidities and association rules.

**Figure 3:**
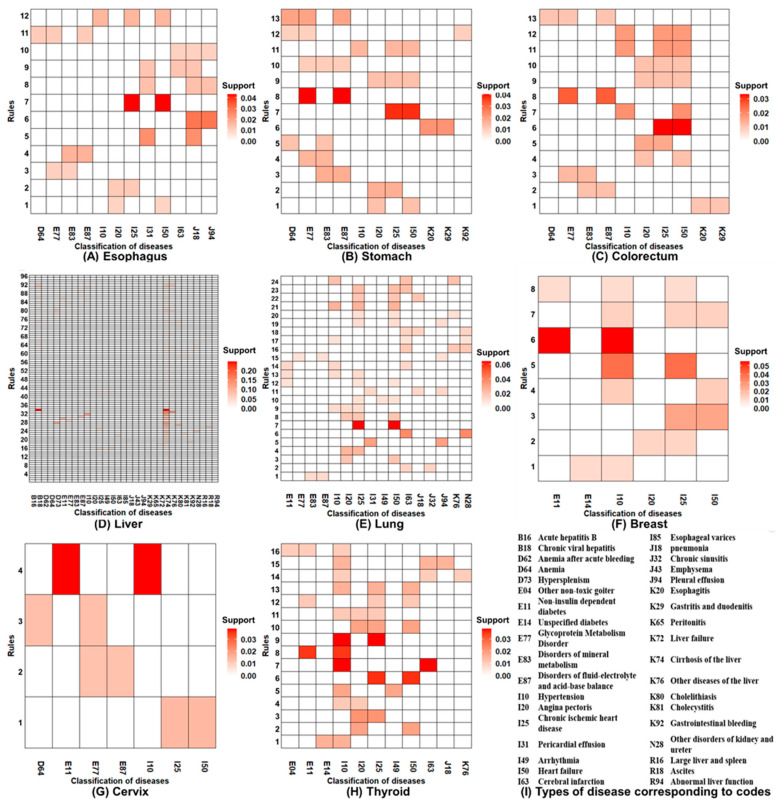
The heatmaps for association rule analyses of comorbidities co-occurring with cancer. (**A**) Esophagus; (**B**) Stomach; (**C**) Colorectum; (**D**) Liver; (**E**) Lung; (**F**) Breast; (**G**) Cervix; (**H**) Thyroid; (**I**) Types of disease corresponding to codes.

Figure 3 presents the heatmaps for association rules analyses of comorbidities co-occurring with cancer. Each row represents a different itemset of comorbidities. Each column represents a cancer-related comorbidity. Red represents the support of the itemset of co-morbid diseases; the darker the color, the higher the support. Using esophagus cancer as an example, there were seven dyads and five triads of comorbidities, with a total of 12 diseases. The most common dyad was chronic ischemic heart disease (I25) and heart failure (I50), and the most common triad was hypertension (I10), chronic ischemic heart disease (I25), and heart failure (I50).

Overall, cancers were strongly associated with cardiovascular diseases and metabolic syndrome in elderly patients, such as diabetes (E11), glycoprotein metabolism disorder (E77), mineral metabolism disorder (E83), disorder of fluids and electrolytes and acid-base balance (E87), hypertension (I10), angina pectoris (I20), chronic ischemic heart disease (I25), and heart failure (I50). In addition to cardiovascular and metabolic diseases, anemia (D64), and digestive system diseases (e.g., gastrointestinal bleeding (K92), other diseases of the liver (K76), esophagitis (K20), and gastritis and duodenitis (K29)) were common comorbidities in elderly patients with cancer, especially with respect to liver, stomach, and colorectal cancers.

Liver cancer involved the largest number of types of co-morbid diseases and association rules, with a total of 31 diseases and 96 rules. The categories of these diseases were as follows: infectious diseases; blood and hematopoietic organ diseases; metabolic diseases; cardiovascular diseases; respiratory diseases; digestive system diseases; genitourinary system diseases; and symptoms, signs, and ill-defined conditions. Among the categories of disease, the rule with the highest degree of support was chronic viral hepatitis (B18) and liver cirrhosis (K74) (support, 24.77%). Although the types of comorbid diseases involved in lung cancer were less than liver cancer, the types of comorbid diseases involved in lung cancer were greater in number than other cancers (17 diseases and 24 rules). The categories of these diseases included metabolic, cardiovascular, respiratory, digestive, and reproductive system diseases. The rule with the highest degree of support was chronic ischemic heart disease (I25) and heart failure (I50) (support, 6.45%).

In addition to cardiovascular and metabolic diseases, common comorbidities of stomach and colorectal cancers were digestive tract inflammation, including esophagitis (K20), gastritis, and duodenitis (K29), while the common comorbidities of esophagus cancer were pneumonia (J18) and pleural effusion (J94). Cervix and breast cancers, cancers of the female reproductive system, had fewer types of comorbidities (mainly common cardiovascular and metabolic diseases).

## 4. Discussion

This study was conducted to analyze the association between various types of cancers and comorbid diseases. Diagnostic data used in this study were collected from hospital discharge medical records distributed throughout Jilin province, providing data from a diverse population to comprehensively examine and characterize wide-ranging patterns of comorbidities. We used ARM to identify common comorbidities and comorbidity patterns in patients with cancer. These influential comorbidities could be targeted for specific intervention and/or screening.

The results of the high PRs and support for blood system diseases and digestive system diseases showed a strong association between blood and digestive system diseases and cancer, and there was a higher risk of comorbidities in cancer patients than non-cancer patients. Anemia is a common issue in cancer patients, which has several possible causes and contributing mechanisms. Anemia may be the result of the cancer itself, cancer treatment, blood losses, hemolysis or inflammatory cytokines associated with chronic disease [30]. For example, some elements related to hemoglobin synthesis, such as iron, cannot be fully utilized, or endogenous erythropoietin (EPO) is relatively insufficient [31,32,33]. Inflammation is often associated with the development and progression of cancer [34]. Digestive system inflammation can induce carcinogenic mutations, increase the risk of cancers, and promote cancers initiation [35].

ARM analysis showed that cardiovascular and metabolic diseases have a high degree of support. In addition, ARM analysis showed high support rules between cardiovascular and metabolic diseases and each type of cancer, which are consistent with other research results [18,36,37]. Compared with non-cancer patients, the PRs of cancer patients were significantly less than one, which showed that the support scores of cardiovascular and metabolic diseases were highly correlated with prevalence. Diabetes, hypertension, and heart failure are the most common chronic diseases in elderly patients, and have a high prevalence and strong associations [38]. Because of the high incidence of diabetes, hypertension, and coronary heart disease, the possibility of complications with other diseases in the population are increased and the comorbid relationship between cardiovascular disease and cancer may be overestimated [25].

Our study has also shown that different cancer sites have different comorbidity patterns. The study found that liver and lung cancers had the most types and rules of complications, followed by gastrointestinal cancers, such as esophagus, gastric, and colorectal cancers. In contrast, cervical and breast cancer, cancers of the female reproductive system, had fewer comorbidities, which is in agreement with published results [21]. Therefore, we take the comorbidity model of liver cancer as an example to discuss the relationship between comorbidities and cancer. The results showed a strong association between liver cancer, cirrhosis and chronic viral hepatitis. The most common causes for liver cancer are chronic viral hepatitis B and C infection [39], and cirrhosis is a strong risk factor for liver cancer [40]. Liver cancer is frequently accompanied by one or more components of metabolic diseases, because metabolism is the most important function of the liver. The metabolism of sugar, protein, fat, vitamins and electrolytes is closely related to the liver. Liver lesions occur in patients with liver cancer, leading to metabolic disorders [41,42]. This finding indicates that elderly patients with liver cancer face a higher risk of comorbidity diseases. It is recommended that elderly patients with liver cancer pay more attention to co-morbid diseases and strengthen the prevention and management of co-morbid diseases.

There were several limitations to this study. We can only identify diseases that were coded during hospitalization, and there is limited information about the onset of comorbidities, so we cannot determine the precedence or causality of comorbidities. Moreover, the threshold set of association rules is empirical, and the results may vary depending on the selected threshold. Due to space limitations, only rules and comorbidities with high reliability levels were analyzed. Therefore, we will explore the mechanisms underlying comorbidities and the rules of occurrence and development of comorbidity patterns in more detail in subsequent studies.

## 5. Conclusions

This study used ARM from hospital discharge data to identify an extensive list of important comorbidities in cancer patients. Our work demonstrates how clinically derived data can be used to identify cancer-related comorbidities and the ARM algorithm can be used to analyze the comorbidities associated with cancer. This method may be widely applied to exploring other chronic disease-related comorbidities. From the overall pattern of comorbidities, cardiovascular disease, metabolic disease, anemia, and digestive system disease were the most common comorbidities in elderly patients with cancer. Studies have also shown that different cancer sites have different comorbidity patterns compared with other cancer sites. Elderly patients with liver cancer face the highest risk of comorbidities. These results can provide references for the clinical diagnosis and active prevention of cancer comorbidities, and play a positive role in improving the quality of life of patients with cancer comorbidities.

## Figures and Tables

**Table 1:** Number of comorbidities of patients with and without cancer.

Variables	Cancers									Without Cancer (*n* = 80,574)
	All (*n* = 80,574)	Esophagus (*n* = 3088)	Stomach (*n* = 8220)	Colorectum (*n* = 16,961)	Liver (*n* = 8710)	Lung (*n* = 28,282)	Breast (*n* = 11,231)	Cervix (*n* = 2623)	Thyroid (*n* = 1459)	
All	3 [1,2,3,4,5,6]	2 [1,2,3,4,5]	2 [1,2,3,4,5]	2 [1,2,3,4,5]	4 [2,3,4,5,6]	4 [2,3,4,5,6,7]	2 [1,2,3,4]	2 [1,2,3,4]	2 [1,2,3,4]	2 [1,2,3,4]
By gender										
Male	3 [1,2,3,4,5,6]	2 [1,2,3,4,5]	2 [1,2,3,4,5]	2 [1,2,3,4,5]	4 [2,3,4,5,6]	4 [2,3,4,5,6,7]	——	——	2 [1,2,3,4,5]	2 [1,2,3,4]
Female	3 [1,2,3,4,5]	3 [1,2,3,4,5]	3 [1,2,3,4,5]	3 [1,2,3,4,5]	4 [2,3,4,5,6]	4 [2,3,4,5,6,7]	2 [1,2,3,4]	2 [1,2,3,4]	2 [1,2,3,4]	2 [1,2,3,4,5]
By age										
60–70	3 [1,2,3,4,5]	2 [1,2,3,4,5]	2 [1,2,3,4]	2 [1,2,3,4,5]	4 [2,3,4,5,6]	4 [2,3,4,5,6,7]	2 [1,2,3,4]	2 [1,2,3]	2 [1,2,3,4]	2 [1,2,3,4]
70–80	3 [1,2,3,4,5,6]	2 [1,2,3,4,5]	3 [1,2,3,4,5]	3 [1,2,3,4,5]	4 [2,3,4,5,6]	4 [2,3,4,5,6,7]	3 [1,2,3,4,5,6]	2 [1,2,3,4]	2 [1,2,3,4,5]	2 [1,2,3,4,5]
≥80	4 [2,3,4,5,6,7,8]	3 [1,2,3,4,5]	4 [2,3,4,5,6,7]	4 [1,2,3,4,5,6,7]	5 [2,3,4,5,6,7,8]	5 [2,3,4,5,6,7,8]	4 [1,2,3,4,5,6]	4 [2,3,4,5]	5 [2,3,4,5,6,7,8]	3 [1,2,3,4,5]

Number of comorbidities presented as median (interquartile range).

**Table 2:** Comorbidity prevalence and prevalence ratios in elderly patients with and without cancer.

Comorbid Disease Categories	ICD-10 Codes	Patients with Cancer *n* (%)	Patients without Cancer *n* (%)	PRs(95%CI)	*p* Value
Infectious diseases	A00-B99	5501(6.79)	1823(2.26)	3.00(2.85–3.16)	<0.001
Blood system diseases	D50-D89	8037(9.92)	3913(4.86)	2.04(1.97–2.12)	<0.001
Metabolic diseases	E00-E89	19,617(24.20)	29,110(36.13)	0.67(0.66–0.68)	<0.001
Mental, Behavioral and Neurodevelopmental disorders	F01-F99	58(0.07)	388(0.48)	0.15(0.11–0.20)	<0.001
Diseases of the nervous system	G00-G99	1354(1.67)	3920(4.87)	0.34(0.32–0.37)	<0.001
Diseases of the eye, adnexa, ear and mastoid process	H00-H95	238(0.29)	4143(5.14)	0.08(0.07–0.09)	<0.001
Cardiovascular diseases	I00-I99	28,457(35.11)	46,245(57.39)	0.61(0.61–0.62)	<0.001
Diseases of the respiratory system	J00-J99	19,083(23.54)	12,548(15.57)	1.51(1.48–1.54)	<0.001
Diseases of the digestive system	K00-K95	23,992(29.60)	13,421(16.66)	1.78(1.74–1.81)	<0.001
Diseases of the skin and subcutaneous tissue	L00-L99	455(0.56)	884(1.10)	0.51(0.46–0.57)	<0.001
Diseases of the musculoskeletal system	M00-M99	2283(2.82)	5685(7.06)	0.40(0.38–0.42)	<0.001
Diseases of the genitourinary system	N00-N99	9705(11.97)	9476(11.76)	1.02(0.99–1.05)	0.185
Congenital anomalies	Q00-Q99	343(0.42)	658(0.82)	0.52(0.46–0.59)	<0.001
Symptoms, signs and ill-defined conditions	R00-R99	7931(9.79)	6191(7.68)	1.27(1.23–1.32)	<0.001
Injury, poisoning and certain other consequences of external causes	S00-T88	620(0.76)	2429(3.01)	0.25(0.23–0.28)	<0.001

PR, prevalence ratio. CI, confidence intervals. The code number of each system was found at https://icd.who.int/browse10/2010/en.

**Table 3:** Prevalence ratios of co-morbid diseases in elderly patients with specific cancers.

Comorbid Disease Categories	Esophagus PR (95% CI)	Stomach PR (95% CI)	Colorectum PR (95% CI)	Liver PR (95% CI)	Lung PR (95% CI)	Breast PR (95% CI)	Cervix PR (95% CI)	Thyroid PR (95% CI)
Infectious diseases	1.23(1.00–1.52)	1.25(1.10–1.43) *	1.30(1.18–1.44) *	16.85(15.98–17.76) *	1.70(1.58–1.83) *	0.70(0.61–0.82) *	1.15(0.90–1.46)	0.58(0.37–0.90)
Blood system diseases	1.69(1.49–1.91) *	3.29(3.11–3.49) *	1.83(1.73–1.94) *	3.66(3.46–3.86) *	1.84(1.75–1.93) *	0.93(0.85–1.02)	2.61(2.35–2.90) *	0.54(0.39–0.74) *
Metabolic diseases	0.55(0.51–0.59) *	0.64(0.62–0.67) *	0.58(0.56–0.60) *	0.78(0.76–0.81) *	0.73(0.72–0.75) *	0.56(0.54–0.59) *	0.58(0.54–0.63) *	1.29(1.22–1.36) *
Mental, behavioral and neurodevelopmental disorders	0.13(0.03–0.54) *	0.13(0.05–0.31) *	0.15(0.08–0.26) *	0.12(0.05–0.29) *	0.17(0.11–0.26) *	0.13(0.06–0.27) *	0.18(0.05–0.74)	0.14(0.02–1.01)
Diseases of the nervous system	0.25(0.18–0.34) *	0.29(0.24–0.34) *	0.25(0.22–0.29) *	0.25(0.21–0.30) *	0.51(0.47–0.55) *	0.17(0.14–0.21) *	0.17(0.11–0.25) *	0.21(0.13–0.35) *
Diseases of the eye, adnexa, ear and mastoid process	0.04(0.02–0.09) *	0.07(0.05–0.10) *	0.06(0.04–0.07) *	0.09(0.07–0.13) *	0.08(0.07–0.10) *	0.11(0.08–0.14) *	0.03(0.01–0.08) *	0.15(0.08–0.26) *
Cardiovascular diseases	0.56(0.53–0.59) *	0.46(0.44–0.48) *	0.49(0.48–0.50) *	0.55(0.53–0.57) *	0.81(0.80–0.82) *	0.52(0.51–0.54) *	0.44(0.41–0.47) *	0.53(0.49–0.57) *
Diseases of the respiratory system	1.61(1.52–1.72) *	0.93(0.88–0.98)	0.76(0.72–0.79) *	0.89(0.84–0.94) *	2.84(2.78–2.90) *	0.55(0.51–0.58) *	0.32(0.27–0.38) *	0.99(0.87–1.11)
Diseases of the digestive system	1.49(1.40–1.59) *	2.09(2.03–2.16) *	2.17(2.12–2.23) *	4.14(4.05–4.23) *	1.28(1.24–1.31) *	0.90(0.86–0.94) *	0.73(0.65–0.81) *	0.68(0.58–0.78) *
Diseases of the skin and subcutaneous tissue	0.30(0.16–0.55) *	0.49(0.36–0.66) *	0.55(0.45–0.67) *	0.68(0.53–0.87)	0.58(0.50–0.68) *	0.34(0.25–0.46) *	0.17(0.07–0.42) *	0.31(0.13–0.75)
Diseases of the musculoskeletal system	0.21(0.16–0.28) *	0.21(0.18–0.25) *	0.30(0.27–0.33) *	0.30(0.26–0.35) *	0.55(0.51–0.58) *	0.44(0.39–0.49) *	0.22(0.16–0.30) *	0.66(0.52–0.83) *
Diseases of the genitourinary system	0.84(0.76–0.94)	1.05(0.99–1.11)	1.08(1.04–1.13) *	1.28(1.21–1.35) *	1.13(1.09–1.17) *	0.52(0.49–0.56) *	1.12(1.02–1.24)	0.53(0.43–0.65) *
Congenital anomalies	0.63(0.39–1.04)	0.69(0.51–0.92)	0.59(0.47–0.74) *	0.72(0.54–0.95)	0.42(0.34–0.52) *	0.39(0.28–0.55) *	0.14(0.05–0.44) *	0.84(0.45–1.56)
Symptoms, signs and ill-defined conditions	1.36(1.22–1.51) *	1.48(1.38–1.58) *	1.09(1.04–1.16) *	2.31(2.19–2.43) *	1.33(1.27–1.38) *	0.63(0.58–0.69) *	0.72(0.61–0.84) *	0.85(0.70–1.03)
Injury, poisoning and certain other consequences of external causes	0.27(0.18–0.40) *	0.20(0.15–0.26) *	0.35(0.30–0.41) *	0.19(0.14–0.25) *	0.28(0.25–0.32) *	0.15(0.11–0.20) *	0.24(0.15–0.38) *	0.07(0.02–0.21) *

* = *p* < 0.001.
